# Human mesenchymal stem cell sheets in xeno-free media for possible allogenic applications

**DOI:** 10.1038/s41598-019-50430-7

**Published:** 2019-10-08

**Authors:** Kyungsook Kim, Sophia Bou-Ghannam, Hallie Thorp, David W. Grainger, Teruo Okano

**Affiliations:** 1 Cell Sheet Tissue Engineering Center (CSTEC), Department of Pharmaceutics and Pharmaceutical Chemistry, Health Sciences, University of Utah, 30 South 2000 East, Salt Lake City, Utah 84112 USA; 2 Department of Biomedical Engineering, University of Utah, 36 S Wasatch Dr, Salt Lake City, Utah 84112 USA; 3 Institute of Advanced Biomedical Engineering and Science, Tokyo Women’s Medical University, 8-1 Kawada-cho, Shinjuku-ku, Tokyo 162-8666 Japan

**Keywords:** Regenerative medicine, Tissue engineering, Mesenchymal stem cells

## Abstract

Cell-based therapies are increasingly focused on allogeneic stem cell sources because of several advantages in eliminating donor variability (e.g., aging and disease pathophysiology) affecting stem cell quality and in cell-banked sourcing of healthy donors to enable “off-the-shelf” products. However, allogeneic cell therapy is limited by host patient immunologic competence and inconsistent performance due to cell delivery methods. To address allogeneic cell therapy limitations, this study developed a new allogeneic stem cell sheet using human umbilical cord mesenchymal stem cells (hUC-MSC) that present low antigenicity (i.e., major histocompatibility complex, MHC). Optimal conditions including cell density, passage number, and culture time were examined to fabricate reliable hUC-MSC sheets. MHC II antigens correlated to alloimmune rejection were barely expressed in hUC-MSC sheets compared to other comparator MSC sheets (hBMSC and hADSC). hUC-MSC sheets easily graft spontaneously onto subcutaneous tissue in immune-deficient mice within 10 minutes of placement. No sutures are required to secure sheets to tissue because sheet extracellular matrix (ECM) actively facilitates cell-target tissue adhesion. At 10 days post-transplantation, hUC-MSC sheets remain on ectopic target tissue sites and exhibit new blood vessel formation. Furthermore, implanted hUC-MSC sheets secrete human HGF continuously to the murine target tissue. hUC-MSC sheets described here should provide new insights for improving allogenic cell-based therapies.

## Introduction

Mesenchymal stem cells have been an interest for allogeneic cell-based therapies for decades^1,2^. Nearly 500 clinical trials using mesenchymal stem cell (MSC) therapies (http://www.clinicaltrial.gov/) have treated over 2000 patients to date^2^. Many of these involve intravenous infusions of either autologous or allogenic MSCs as cell suspensions. Therapeutic benefits from any of these trials is arguably marginal to date, despite reasonable preclinical evidence. Consensus on mechanisms for MSC cell therapy does not currently exist. Nonetheless, several hypotheses have been forward to explain observed MSC clinical benefits^3^, specifically, their intrinsic ability to (1) differentiate into diverse and distinct cell lineages, (2) produce an array of soluble bioactive factors central to cell maintenance, survival and proliferation, (3) modulate host immune responses, and (4) migrate as recruited to sites of injury to mitigate damage and promote healing (i.e., homing)^2^. In certain reported cases, MSCs seemingly avoid allogeneic rejection in humans and in animal models^4–8^. For these reasons, MSCs have frequently been used to treat various diseases such as myocardial infarcts, graft-versus-host disease, Crohn’s Disease, cartilage and meniscus repair, stroke, and spinal cord injury^2,9–11^. This produces realistic possibilities for pioneering allogeneic cell therapies that, as off-the-shelf products, might someday side-step the unfavorable costs and development disincentives associated with autologous stem cell treatment paradigms^12^.

More practically, allogeneic cell sources must be able to demonstrate their reliable capabilities to elicit meaningful therapies under standard immunologic competence in host patient allogeneic tissues. This includes reliable cell homing to and fractional dose engraftment or retention for sufficient duration at the tissue site of therapeutic interest^13^. Current estimates are that less than 3% of injected stem cells are retained in damaged myocardium 3 days post-injection following ischemic injury^14^. Additionally, most administered cells that engraft into target tissue will die within the first few weeks^15^. Effective translation of MSC therapies is currently hindered by the clinical inability to target these therapeutic cells to tissues of interest with reasonable efficiency and significant engraftment and retention. Conventional MSC therapies are injectable cell suspensions, often derived from culture-adherent cells harvested from culture plastics using proteolytic enzymes. Proteolyzed, dissociated cells require substantial time to recover from harvest, suspension and loss of cell-cell junctions, associated matrix and cell receptors. MSCs maintained in two-dimensional (2D) culture systems are shown to gradually lose intrinsic proliferative potential, colony-forming efficiency, and differentiation capacity over time^16–18^. Additionally, MSC homing to target tissue areas are compromised because intrinsic MSC adhesion components and mechanisms are damaged by proteolytic enzyme treatment^19,20^. Integrating healing physiology and regenerative potential is reduced by low cell retention and engraftment into target tissues and organs, a key factor in successful cell therapy^21^.

Human umbilical cord-derived MSCs (hUC-MSCs) used in this study represent a promising allogeneic cell source for stem cell therapy among diverse MSC types, with increasing clinical evidence^22–25^. hUC-MSCs exhibit low HLA expression and higher paracrine effects compared to human bone marrow stem cells (hBM-MSC)^22,26,27^. Furthermore, intravenously infused allogenic hUC-MSC treatments induced no adverse host immune responses and produced clinically significant improvements in patients either with heart failure, with spinal cord, or with multiple sclerosis^22–25^. Despite these optimistic early results, cell delivery and engraftment must be improved because few injected cells reach target tissue sites with sufficiently long retention or viability to enact reliable therapeutic effects.

**Figure 1:**
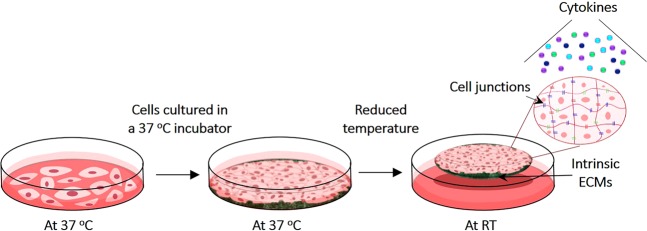
Cell sheet experimental protocol. Human umbilical cord stem cells (hUC-MSCs) were seeded on temperature responsive cell culture dishes (TRCD) and cultured to confluence in a 37 °C cell culture incubator. Cultured cells were detached from TRCDs as intact cell sheets within 20 min at room temperature (RT), preserving their functional structures such as extracellular matrixes (ECMs) and cell-cell-junctions and cytokine secretion ability without using proteolytic enzyme treatments.

Okano and colleagues previously developed a versatile cell delivery method exploiting new cell culture capabilities from temperature-responsive cell culture dishes (TRCD)^28,29^. These polymer-grafted tissue culture surfaces release cultured cells as confluent living sheets in response to small changes in culture temperature, notably without enzymes. Recovered cell sheets completely retain native forms, confluent phenotypes and organization, cell-cell communication, intact extracellular matrix (ECM) and tissue–like behaviors (Fig. 1)^20,30,31^. Moreover, intact ECM decorating cell sheets serves as a natural tissue adhesive, eliminating needs to suture. This allows cell sheets to rapidly and spontaneously attach to target tissues. This feature increases their engraftment efficiency^30,32,33^. Using this cell sheet technology and autologous patient cell sources, Okano and colleagues have treated several human diseases in clinical trials^33–43^. Motivated by the compelling need to develop allogeneic cell sheets for more widespread use of such therapies, MSC sheets have been proposed^30,44,45^. This study aimed to develop and characterize a potential allogeneically acceptable hUC-MSC sheet. Towards this goal, baseline criteria to prepare hUC-MSC sheets were optimized. hUC-MSC sheets fabricated using this strategy were evaluated both structurally and functionally.

## Results

### hUC-MSC sheet preparation by varying initial cell seeding and passage numbers

**Figure 2:**
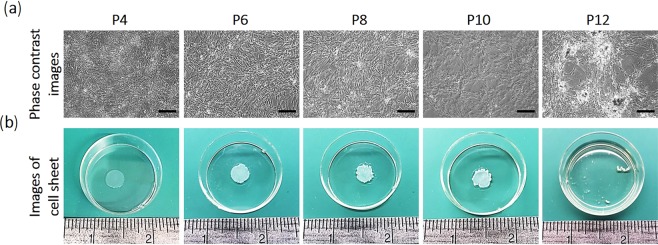
Passaging influence. hUC-MSC sheet morphological observations using cell passages 4, 6, 8, 10 and 12 seeded at 3.2–4 × 10^3^ cells/cm^2^. (**a**) Morphology of passage 4, 6, 8, 10 and 12 cells observed using phase-contrast microscopy before sheet detachment. (**b**) Successful fabrication of hUC-MSC sheets using passage 4, 6, 8 and 10 cells. In contrast, passage 12 cells detached as non-contiguous disconnected cellular structures. Scale bars = 100 μm.

hUC-MSCs were cultured on flasks and sub-cultured using trypsin every 5 days from passages 4 to 12 (Table 1). Cells were proliferated 16–20 times from initial cell seeding numbers between passages 4–8 during sub-culture. However, cell proliferation rates dramatically decrease from passage 9. Cell numbers were 14, 10.9, 7.5, and 3.1-fold increased from initial cell seeding numbers at passage 9, 10, 11, and 12, respectively. Cells in passage 10 required one day more to reach confluence and yield cell sheets than cells in passages 4–8 at the same seeding density (Fig. 2a,b). Cells in passage 12 exhibited heterogeneously cultured morphologies, lost contact inhibition, and clumped into multi-layered aggregates rather than consistent monolayers (Fig. 2a). When culture temperature was reduced to RT, cells in passage 12 detached from TRCD, but not as sheets, as recovered from passages 4–10 (Fig. 2b). Passage 12 cells were not able to form stable sheets due to reduced cell proliferation rates and inadequate cell-cell junction formation after increased passaging (Fig. S1 and Table 1). Cells should therefore be used from passages 4 to 8 to produce consistent cell sheet quality.

**Table 1:** Growth rates for hUC-MSCs in passages 4–12.

	Cell viability	Initial cell seeding density	Expanded cell density	Fold increase
Passage 4	96.0%	4.0 × 10^3^/cm^2^	6.5 × 10^4^/cm^2^	16.3
Passage 5	96.0%	3.5 × 10^3^/cm^2^	6.7 × 10^4^/cm^2^	19.0
Passage 6	97.5%	3.2 × 10^3^/cm^2^	5.5 × 10^4^/cm^2^	17.2
Passage 7	96.2%	3.2 × 10^3^/cm^2^	5.5 × 10^4^/cm^2^	17.3
Passage 8	96.7%	3.2 × 10^3^/cm^2^	6.6 × 10^4^/cm^2^	20.7
Passage 9	98.5%	3.2 × 10^3^/cm^2^	4.5 × 10^4^/cm^2^	14.0
Passage 10	97.6%	3.2 × 10^3^/cm^2^	3.5 × 10^4^/cm^2^	10.9
Passage 11	97.4%	3.2 × 10^3^/cm^2^	2.4 × 10^4^/cm^2^	7.5
Passage 12	97.4%	3.2 × 10^3^/cm^2^	1.0 × 10^4^/cm^2^	3.1

**Figure 3:**
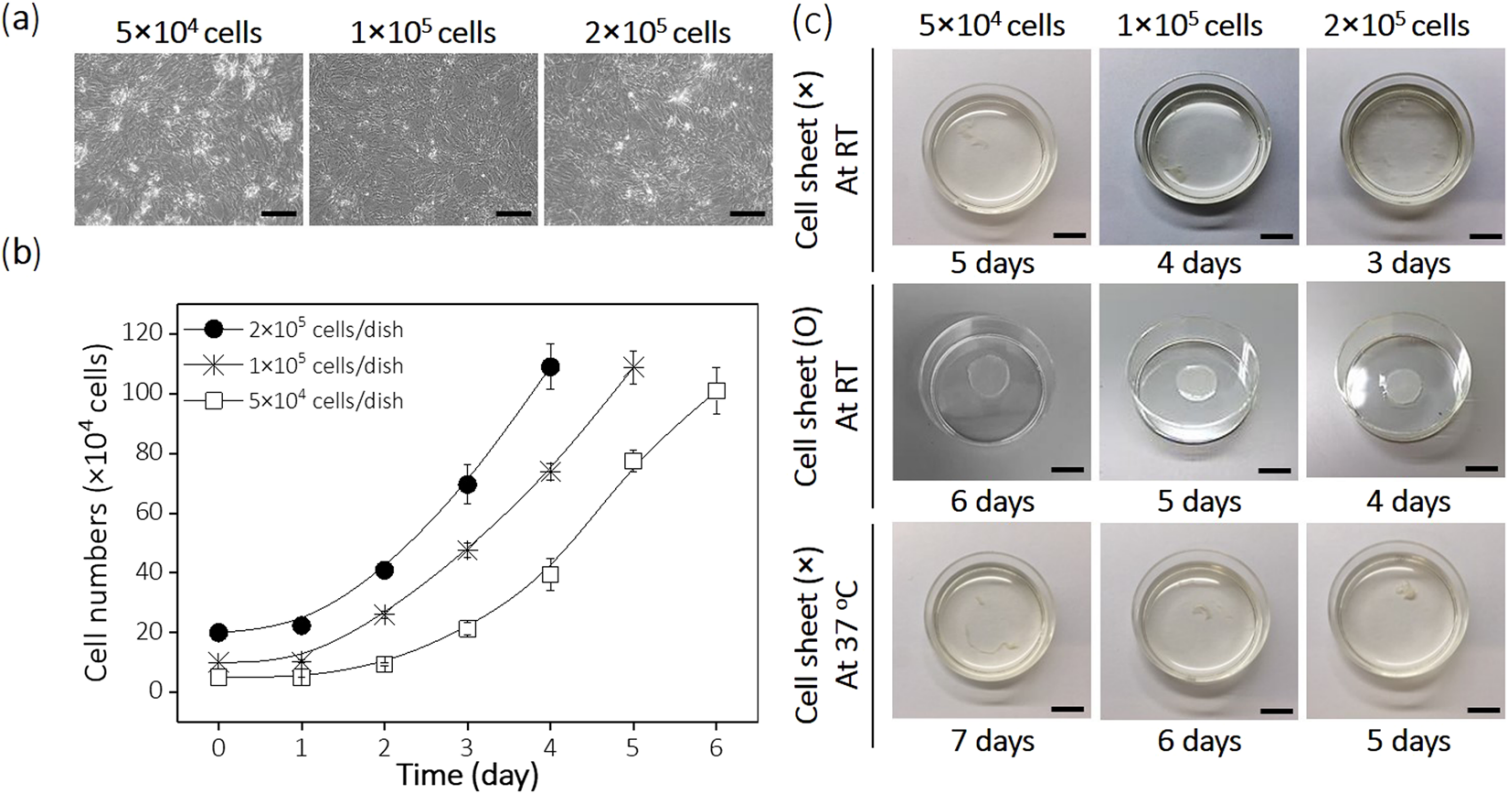
Cell seeding influence. Morphological observation, cell proliferation rate, and cell sheet fabrication for hUC-MSCs seeded at 5 × 10^4^, 1 × 10^5^ and 2 × 10^5^ initial cell numbers. (**a**) Cells cultured on TRCD and observed prior to sheet detachment. (**b**) Cell numbers counted using a hemacytometer after cells were seeded on TRCD until hUC-MSC sheets are observed. (**c**) Cells detached as disconnected pieces at one day prior to confluence at 3, 4 and 5 days in 2 × 10^5^, 1 × 10^5^ and 5 × 10^4^ initial cell seeded groups, respectively. Intact cell sheets were successfully fabricated at 4, 5 and 6 day for seeding densities of 2 × 10^5^, 1 × 10^5^ and 5 × 10^4^ initial cell number groups, respectively. One day post-confluence, cultured cells spontaneously detach as aggregated forms without TRCD temperature changes at 5, 6 and 7 days for the 2 × 10^5^, 1 × 10^5^ and 5 × 10^4^ initial cell seeded groups, respectively. Scale bars indicate 100 μm in (**a**). Scale bars indicate 1 cm in (**c**).

Initially seeded cell numbers of 5 × 10^4^, 1 × 10^5^, and 2 × 10^5^ cells/dish in passage 6 reached confluence at 6, 5, and 4 days, respectively (Fig. 3a,b), reliably producing hUC-MSC sheets (Fig. 3c). Cell sheets from all different initial cell number groups spontaneously detached from TRCDs without temperature change (i.e., at 37 °C) at 5, 6, and 7 days in the 2 × 10^5^, 1 × 10^5^, and 5 × 10^4^ seeded cells/dish groups, respectively (Fig. 3c) once cells were over-confluent. One day prior to cell confluence, adherent cells were less than 70–80% confluent and detached as partially broken sheet fragments when temperature was reduced to RT due to insufficient cell density. Recovery of hUC-MSC sheets either one day before or one day after cells reaching confluence on TRCDs was not possible (Fig. 3c). These results indicate that cell sheets must be prepared carefully and recovered at the precise time-point when cells are neither under- or over-confluent in order to detach the cells as a thermally recovered sheet.

### hUC-MSC multi-potency and stem cell surface marker characterization

**Figure 4:**
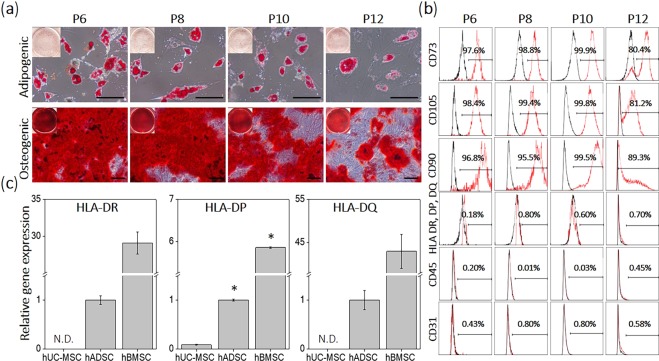
hUC-MSC differentiation potential and stem cell phenotype. hUC-MSCs (passages 6–12) were differentiated into (**a**) adipocytes and osteocytes. Differentiation potential of hUC-MSCs was dramatically decreased as passage numbers increased. hUC-MSCs displayed positive expression of (**b**) CD 73 and negative expression of HLA-DR, DP, DQ consistently from passage 6 to 12. (**c**) HLA-DR, DP, DQ gene expression levels of hUC-MSC sheet were detected by quantitative RT-PCR analysis and compared with other types of MSC sheets (adipose- and bone marrow-derived mesenchymal stem cells) Scale bars in (**a**) represents 20 μm. N.D. represents no detection in (**c**). (**c**) *p < 0.01.

To qualify the cell source, hUC-MSC differentiation potential and stem cell phenotype were characterized for passages 6, 8, 10, and 12. Positive osteogenic and adipogenic differentiation were both observed in induced hUC-MSC cultures from passage 6, 8, 10, and 12 via Alizarin Red and Oil Red O staining, respectively (Fig. 4a). Positive osteogenic and adipogenic differentiation declined from passage 6 through passage 12, with passage 12 cultures showing minimal differentiation. hUC-MSCs expressed a surface phenotype consistent with mesenchymal stem cells from passage 6 to 10, showing positive expression of CD73, CD105, and CD90. Passage 12 cells showed lower positive expression of CD73, CD105 and CD90, compared to passage 6–10 cells (Fig. 4b). hUC-MSCs in all passage numbers were negative for HLA-DR, DP, and DQ, CD45, and CD31 by qPCR. Therefore, hUC-MSC passages 6 should be appropriate to prepare hUC-MSC sheets with higher multi-potency while maintaining stem cell phenotypes.

### Allogeneic immune response of hUC-MSC sheet

To verify MHC II antigen expression of hUC-MSC sheet, quantitative RT-PCR analysis was performed to measure HLA-DR, DP, and DQ gene expression levels of hUC-MSC sheets, compared to other types of MSC sheets (hBMSC and hADSC sheets) at passage 6. HLA-DR and DQ gene expression levels in hUC-MSC sheets were not detectable while hADSC and BMSC sheets clearly exhibited HLA-DR and DQ gene expression. HLA-DP gene expression levels in hUC-MSC sheets were significantly lower than other hBMSC and hADSC sheets (Fig. 4c). These data demonstrate that hUC-MSC sheets lack or bear low MHC II antigens compared to other types of human MSC sheets.

### Structural analysis of hUC-MSC sheets

**Figure 5:**
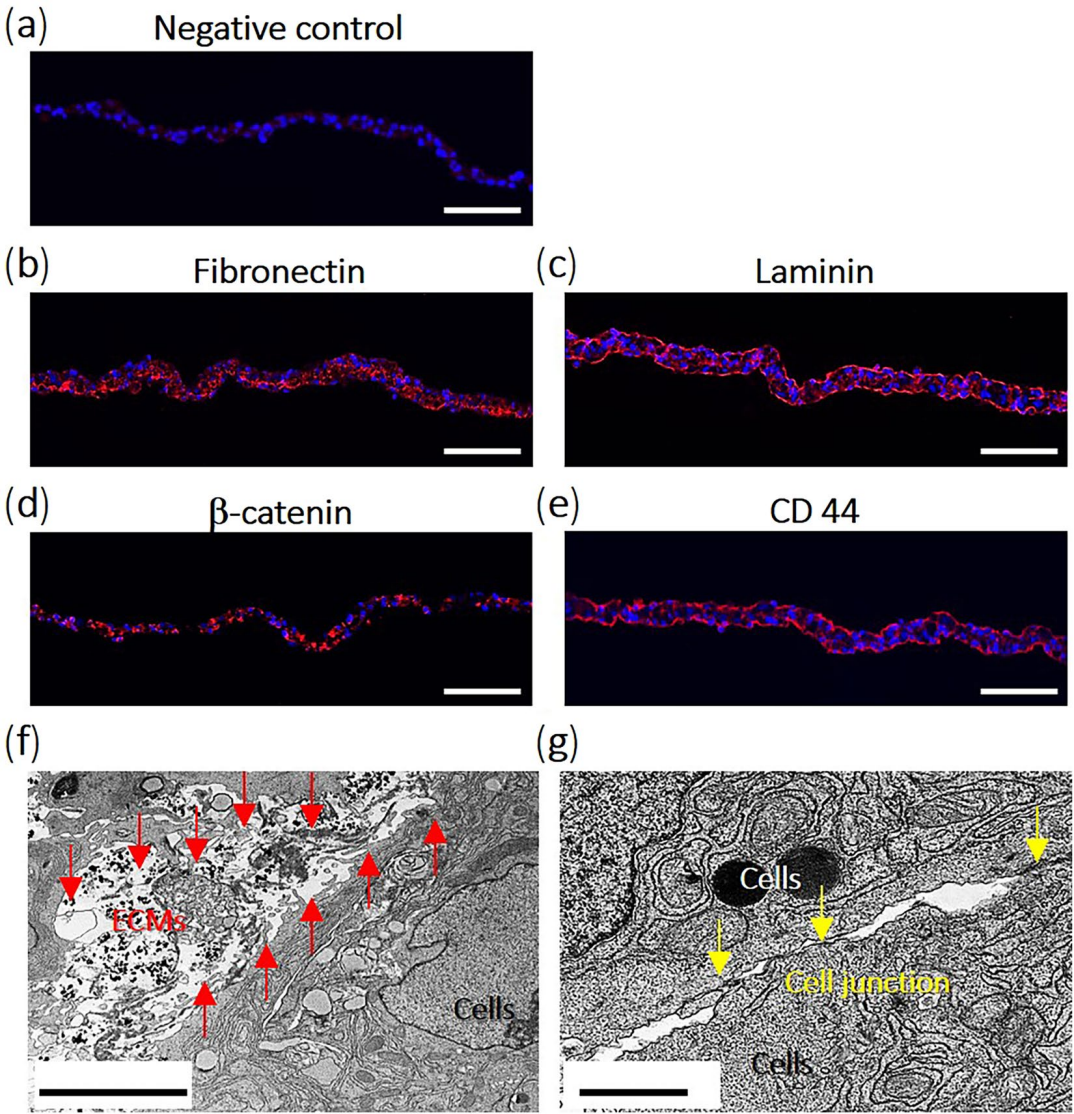
Cell-cell structural analysis using immunohistochemistry (IHC) and transmission electron microscopy (TEM). Cells successfully detached as sheets from TRCD by temperature changes at 4 days after seeding. Cell sheets were stained with isotype negative control antibody (**a**), ECM ((**b**) fibronectin and (**c**) laminin), (**d**) cell junction β-catenin, and (**e**) stem cell surface marker (CD44) antibodies to confirm that cell sheets preserved their functional structures after detachment. In TEM images, hUC-MSC sheets preserved their (**e**) ECMs and (**f**) cell-cell junction structures after detachment. Red arrow = ECMs; yellow arrow = cell junctions in the hUC-MSC sheet. Scale bars indicate 100 μm in (**a**–**d**). Scale bars (**d**,**e**) indicate 5 μm and 1 μm, respectively.

Passage 6 cells were cultured on TRCD for 4 days and resulting cell sheets were recovered from TRCD using temperature reduction to RT. The cell sheet was stained for fibronectin, laminin, β-catenin, and CD44 to verify hUC-MSC sheet retention of functional structures and stem cell phenotypes after sheet detachment. Fibronectin and laminin, important ECM components that promote cell and tissue attachment^46–49^, were strongly expressed across the entire cell sheet surface (Fig. 5b,c) compared to the negative control (Fig. 5a). β-catenin, part of the protein complex forming cell adherent junctions^46^, shows prominent staining between cells (Fig. 5d). CD44-positive expression, an MSC surface marker^50^, was observed on cell surfaces (Fig. 5e). Retention of ECM, cell junction, and MSC surface proteins indicates that select functional proteins produced during culture are preserved after cell sheet harvest. Inter-cellular structures within cell sheets were observed by TEM. Horizontal sectioning showed ECM structures within cell sheets (Fig. 5f), including numerous cell-cell junctions (Fig. 5g). These results suggest that hUC-MSC sheets structurally retain functional proteins related to natural cell functions such as cell communication and cell adhesion.

### Secretion of hepatocyte growth factor (HGF) and tumor necrosis factor-alpha (TNF-α)

**Figure 6:**
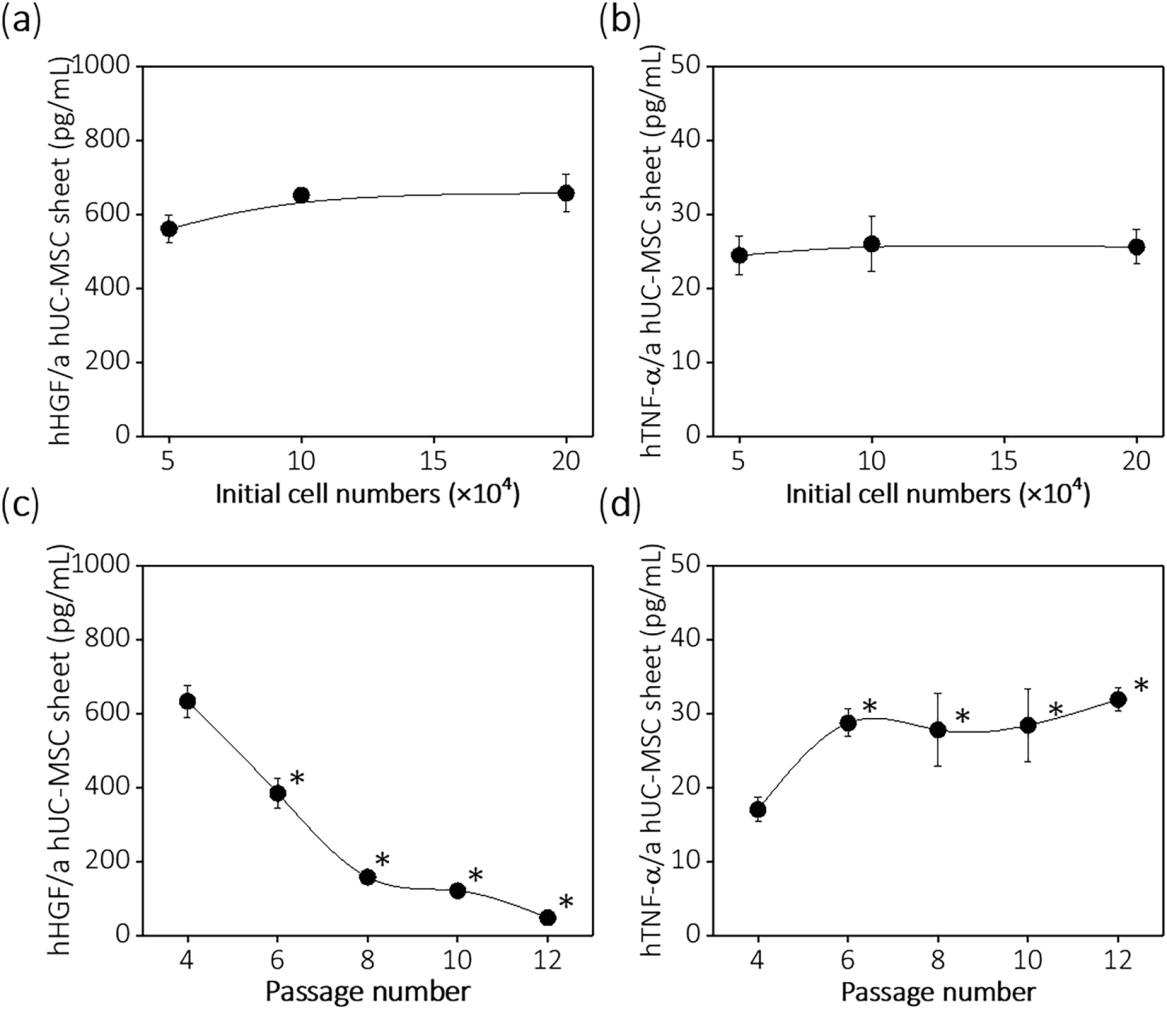
Cytokine analysis of human hepatocyte growth factor (HGF) and tumor necrosis factor-alpha (TNF-α) secreted from hUC-MSC sheets. hHGF (anti-inflammatory cytokine) and hTNF-α (pro-inflammatory cytokine) were detected in culture supernatant for cells cultured for 24 hours. (**a**) no significant differences in hHGF secretion in 2 × 10^5^, 1 × 10^5^, and 5 × 10^4^ initial cell seeded groups. (**b**) hTNF-α barely detected and not significantly different in 2 × 10^5^, 1 × 10^5^ and 5 × 10^4^ initial cell seeded groups. (**c**) Significant reductions in hHGF secreted from hUC-MSC sheets as passage increases. (**d**) hUC-MSC sheet fabricated using passage 4 cells secreted significantly lower amount of hTNF-α, compared to hUC-MSC sheet fabricated using passage 6, 8, 10, and 12 cells *p < 0.05.

Human anti-inflammatory cytokine HGF^51,52^, and pro-inflammatory cytokine TNF-α^53^ secreted from hUC-MSCs in culture supernatant were measured to support paracrine effects of the fabricated hUC-MSC sheets *in vitro*. No significant differences in amounts of hHGF were seen in 2 × 10^5^, 1 × 10^5^, and 5 × 10^4^ cells/dish groups at passage 6 (Fig. 6a). Pro-inflammatory cytokine (hTNF-α) was barely detectable in 2 × 10^5^, 1 × 10^5^, and 5 × 10^4^ cells/dish groups (Fig. 6b). hUC-MSC sheets fabricated using passage 4 cells secreted significantly higher concentrations of hHGF (633 pg/mL), compared to hUC-MSC sheets fabricated using passage 6, 8, 10, and 12 cells. Amounts of hHGF secreted from hUC-MSC sheets dramatically decreased as passage number increased (Fig. 6c). hTNF-α was barely secreted (16–35 pg/mL) from hUC-MSC sheets (Fig. 6d) and hUC-MSC sheets fabricated using passage 4 had significantly lower concentrations of hTNF-α, compared to hUC-MSC sheets fabricated using passage 6, 8, 10, and 12 cells. Results therefore demonstrate that hUC-MSC passage number is important factor to influence resulting hUC-MSC sheet cytokine secretion properties.

### Cell sheet implantation into immune-deficient mice

**Figure 7:**
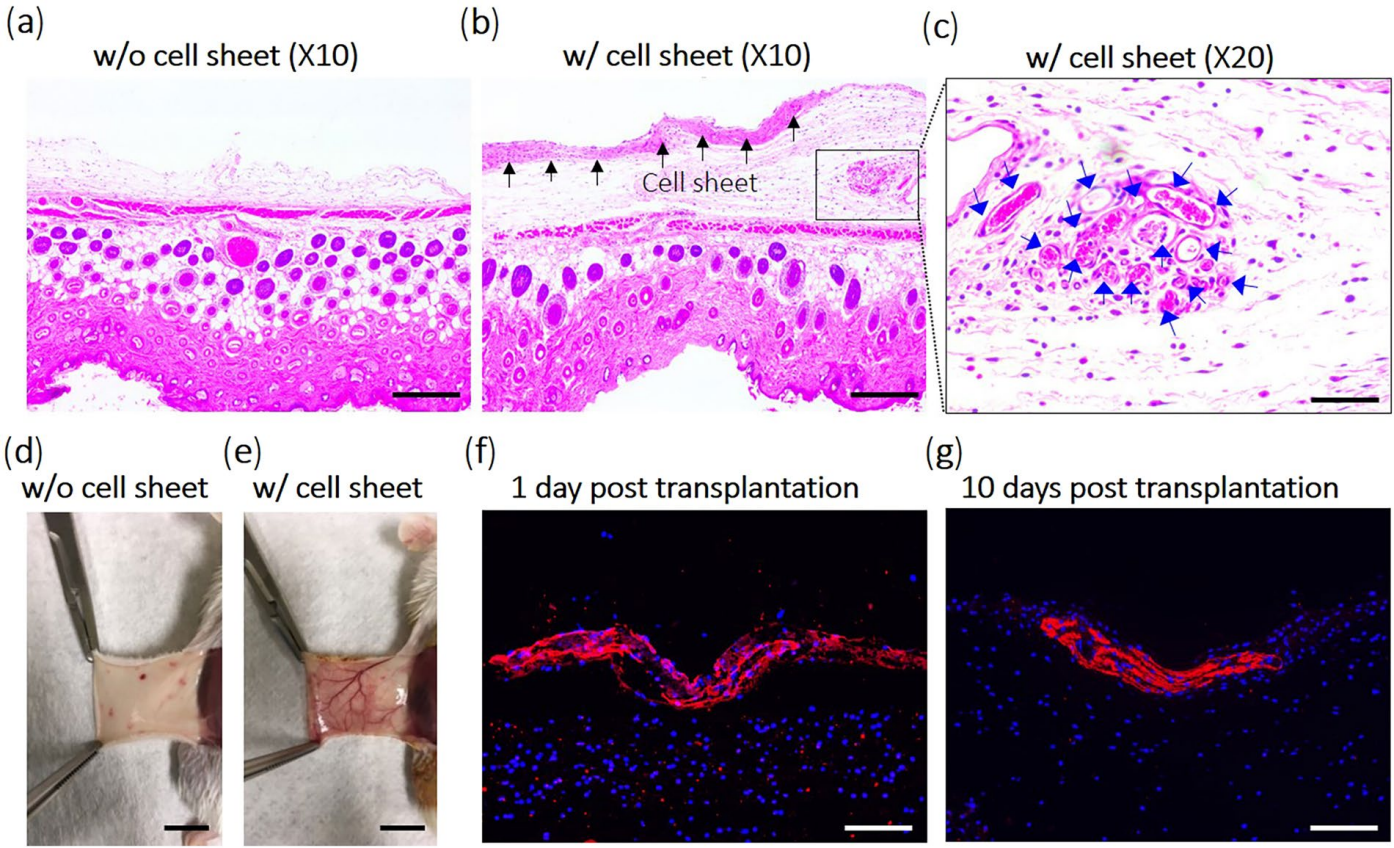
Implanted hUC-MSC sheet retention *in vivo*. hUC-MSC sheets implanted within subcutaneous tissue in immuno-deficient mice. (**d**,**e**) At 10 days after implantation, hUC-MSC transplanted subcutaneous tissue sites were harvested for histological observation. In H&E-stained images, (**b**) hu-MSC cell sheets were confirmed clearly in subcutaneous tissue implant sites compared to (**a**) normal subcutaneous tissue. Additionally, (**c**) abundant vascular structures are observed in cell sheet implanted groups. hUC-MSC sheets secrete human HGF on the transplanted areas at 1 day (**f**) and 10 days (**g**) post implantation. Black arrow = implanted cell sheet; blue arrow = blood vessels. Scale bars (**a**,**b**) and (**c**,**f**,**g**) indicate 100 μm, 50 μm, respectively. Scale bars (**c**,**d**) indicate 0.5 cm.

hUC-MSC sheets were implanted into dorsal subcutaneous pockets in immune-deficient mice for 10 days to demonstrate stability and engraftment *in vivo*. At 10 days post-transplantation, formation of capillaries (angiogenesis) was observed in cell sheet-transplanted tissue, while subcutaneous tissue without cell sheet transplantation showed only a few fine blood vessels (Fig. 7d,e). H&E staining data demonstrated that cell sheets remained localized on the transplanted area for 10 days after transplantation (Fig. 7a,b). In cell sheet-transplanted groups, a large number of blood vessel structures was observed between transplanted cell sheets and host tissue (Fig. 7c). Transplanted hUC-MSC sheets secrete human HGF continuously for 10 days *in vivo*, an important factor for promoting tissue repair (Fig. 7f,g)^54–58^. This indicates that the hUC-MSC sheets are transplantable and able to secret HGF to target tissue continuously *in vivo*.

## Discussion

This study describes preparation and properties for hUC-MSC sheets, motivated by the clinical need for new, improved allogeneic MSC cell therapeutic strategies. Harvested using enzymes that compromise MSC functions and engraftment capabilities, current injected MSC cell suspensions exhibit low tissue retention and survival, hence sub-optimal therapeutic properties^14^. Cell sheets created without enzymes and as living constructs bearing native ECM and cell receptors intact can be physically placed onto tissue sites with highly improved retention and engraftment efficiencies^32^. In a previous study, rat adipose- and rat bone marrow- derived MSC sheets showed therapeutic effects in repairing scarred myocardium after myocardial infarction^30^, wound healing in a rat model of type 2 diabetes^45^, and in preventing pancreatic leakage^44^. This study creates cell sheets using hUC-MSCs already validated for safety and efficacy in human clinical trials as injected suspensions^22–25^, exhibiting higher paracrine activities with lower allogeneic immune rejection, compared to other MSCs^22^. New MSC sheet fabrication using hUC-MSC can be optimized by exploiting specific culture conditions including passage number, culture time, and initial cell seeding density in this study.

Animal-derived growth supplements, such as fetal bovine serum (FBS), have been widely used for MSC expansion in clinical protocols, and currently accepted by FDA and regulatory bodies for this purpose. However, non-human serum utilized for MSC manufacturing may lead to clinical complications^59,60^. Increasingly, xeno-sourced media components are subject to regulatory scrutiny^61–63^. Many approaches have therefore switched MSC culture conditions to human platelet lysate (hPL) media^61–63^. hPL preparations have no standard composition or quality, and diverse sources are reported to increase cell proliferation, differentiation, and immuno-suppressive properties^59,64,65^. Such evidence shows that hPL-containing xeno-free media, while non-standardized, may offer both strategic and performance advantages in clinical-scale MSC expansion towards therapeutic applications. However, cell aggregation using both bone marrow derived (BMSCs) and adipose derived (ADSC) stem cells is reported to occur when cultured in media including 5% hPL^66,67^. In Fig. 2, phase contrast microscopy images showed cells stacked on top of each other and formation of cell aggregates at higher passage numbers. This feature tends to increase as passage number increases, especially for passage 12 cells. Active cell adhesive or coagulation factors in hPL could be involved in aggregation, interrupting homogeneous cell growth and cell sheet fabrication processes. To prepare reproducible hUC-MSC sheets, new types of cell culture media to prevent aggregation should be considered.

Central to these results is the reliable capability to produce a stable, robust monolayer of hUC-MSCs using commercial TRCD grafted with temperature-responsive polymer coatings that facilitates cell harvest using temperature reduction without destructive enzymes^28,29^. This cell sheet technology produces cultured cell recovery with intact native cell-cell organization, cell-cell communication, intact ECMs, and tissue-like phenotypes. Cell sheets recovered from TRCDs using small changes in culture temperature preserve cell surface-associated ECMs (e.g., fibronectin and laminin), and cell-cell junction proteins such as β-catenin (Fig. 5) that play important roles in promoting cell adhesion and paracrine signaling^46–49^. Cell sheets with native morphologies, confluent phenotypes and organization, cell-cell communications, intact extracellular matrix (ECM) and tissue–like behaviors can be readily transferred to target tissues^30,32,33^. Intact ECM in the hUC-MSC sheets served as a natural tissue adhesive, producing spontaneous sheet-tissue surface lamination and eliminating needs for sutures on tissues^20,30,31^. hUC-MSC sheets implanted into subcutaneous tissue sites in immune-deficient mice rapidly and spontaneously attached to subcutaneous tissue surfaces within 10 min. After 10 days *in vivo*, implanted cell sheets remained intact (Fig. 7). MHC class II antigens known to activate the host immune system against allografts^68,69^ must be considered for any off-the-shelf allogenic cell-banked MSC human source. In this regard, hUC-MSC sheets are shown to express considerably low HLA-DR, DP, and DQ (Fig. 4). hUC-MSC sheets fabricated in this study clearly elevate hUC-MSC paracrine benefits without inducing any adverse host inflammatory responses. These new features of hUC-MSC sheets should have considerable value for improving the transplantation efficacy of stem cell therapies.

Tissue regeneration occurs in two contexts: renewal and recruitment of impaired cells to reform damaged organs (structural regeneration), and response to injury by exploiting paracrine and autocrine signals for cell-induced regeneration (functional regeneration)^70^. To regenerate damaged organs, MSCs must be recruited in sufficient numbers and retain a tissue-specific differentiation ability at the target. hUC-MSCs were unable to differentiate sufficiently to chondrocyte phentoypes in Fig. S2. However, hUC-MSCs exhibit both proliferative tendencies and strong differentiation abilities into both adipocyte and osteocyte lineages by passage 10 (Fig. 4). These properties indicate that hUC-MSCs should retain substantial capabilities to replace impaired cells in various tissue sites given proper retention in numbers and phenotypes. Paracrine MSC properties are important in response to injury in tissue regeneration^71–74^. The hUC-MSC is an attractive candidate for regenerative therapy because of its remarkable paracrine action^22,26^. HGF is involved in the mechanism that induces angiogenesis and inhibits fibrosis, and important to tissue repair^54–58^. In a previous study, HGF and VEGF paracrine effects from hUC-MSCs were significantly higher than that of hBMSCs^22^. In this study, after cell sheet fabrication, cell sheets maintained high levels of HGF secretion (Fig. 6). Additionally, passage 4 cells secreted the highest HGF levels in all initial seeding density groups. For disease applications that require paracrine actions to treat, cell sheets fabricated with early passage number cells should prove more effective. Furthermore, human HGF is continuously secreted from implanted hUC-MSC sheets in immune-deficient murine subcutaneous tissue. Neo-vessel formation demonstrated that hUC-MSC sheet implants induced angiogenesis by paracrine action *in vivo*. hTNF-α, a pro-inflmmatory cytokine related to systemic inflammation and hypercalcemia^75,76^ was barely detectable in all groups, including different initial cell seeding and passage numbers.

Overall, hUC-MSC sheets display several beneficial properties for improving allogeneic MSC cell therapy. Results here have determined that (1) specific conditions (cell seeding density, passage number, and culture media) for reliable xeno-free hUC-MSC sheet fabrication are crucial; (2) hUC-MSC sheets preserve important cell functional structures and paracrine effects with low MHC II profiles; (3) hUC-MSC sheet can rapidly adhere to target tissue; and (4) implanted hUC-MSC sheet continually secrete paracrine factors useful to tissue regeneration.

## Conclusions

The simple fabrication method on TRCDs in hPL allows rapid xeno-free production of robust monolayer hUC-MSC sheets, harvested with small temperature changes instead of destructive proteolytic enzymes. Cell sheet production depends on several critical controlled culture variables, including cell seeding density, passage number, and culture time. Given their paracrine effects and low MHC II profile, fabricated xeno-free hUC-MSC sheets represent promising tissue regeneration potential both structurally and functionally *in vitro*. In future studies, the xenogeneic transplantation of hUC-MSC sheets to wild type mice will provide more information regarding hUC-MSC’s immune-modulatory capacity under immune-competent conditions. With reliable topical tissue site placement, long-term retention and survival, and paracrine factor secretion in immune-deficient mice, the hUC-MSC sheet exhibits promising properties for possible future allogeneic cell therapy. However, to enhance reproducibility and simplify the process routine amenable to clinical scaling needs, cell sheet fabrication methods including hPL cell culture media must be further validated. The structure of (double- or triple-) layered cell sheets will be studied as an approach for further enhancing cell sheet function.

## Materials and Methods

### Human umbilical cord stem cell (hUC-MSC) culture

Banked human umbilical cord mesenchymal stem cells isolated from the subepithelial layer of human umbilical cord tissue (Jadi Cell LLC, Miami, USA IRB-35242)^27^ were cultured in xeno-free cell culture media with Dulbecco’s Modified Eagle’s Medium (DMEM) (Life Technologies, CA, USA) supplemented with 10% human platelet lysate (hPL, iBiologics, Phoenix, USA), 1% Glutamax (Life Technologies), 1% MEM NEAA (Life Technologies), 1% penicillin streptomycin (Life Technologies), at 37 °C in a humidified atmosphere with 5% CO_2_ for 5 days. The working cell bank was established in passage 4. hUC-MSCs were expanded from passage 4 to 6 to fabricate hUC-MSC sheets. Cell culture media was changed every two days.

### hUC-MSC proliferation rates

hUC-MSCs were seeded on 35-mm tissue culture plates (TCP) (Corning, USA) at cell numbers of 5 × 10^4^, 1 × 10^5^ and 2 × 10^5^ cells/dish in xeno-free cell culture media. Cells on TCP were dissociated with TrypLE (Gibco, USA) and cell number counted using a hemocytometer at 1, 2, 3, 4, 5 and 6 days. hUC-MSCs were seeded at a cell density of 3.5 × 10^3^/cm^2^ on 175 cm^2^ tissue culture flasks (Corning, USA) and passaged at 5 days with TrypLE after culturing from passage 4 until 12. Cell number was counted each passage using a hemocytometer.

### hUC-MSC differentiation potential

hUC-MSCs were cultured in xeno-free cell culture media for two passages on TCP. At passages 6, 8, 10, and 12, cells were prepared and induced for osteogenic and adipogenic differentiation. For osteogenic differentiation, cells were plated at 5 × 10^3^ cells/cm^2^ in 35 mm TCP dishes in xeno-free cell culture media. When 60% confluent, cells were induced with osteogenic differentiation media containing αMEM, 10 nM dexamethasone, 82 µg/mL ascorbic acid 2-phosphate, 10 mM β-glycerolphosphate (Sigma-Aldrich). Cells were cultured in osteogenic media at 37 °C for 21 days with media changed every 3 days. To detect positive differentiation, cells were fixed with cold 4% paraformaldehyde (PFA) for 12 minutes and stained with Alizarin Red S- (Sigma-Aldrich) using standard protocols. For adipogenic differentiation, cells were plated at 1 × 10^4^ cells/cm^2^ in 35 mm TCP dishes in xeno-free cell culture media. When 80% confluent, cells were induced with adipogenic differentiation media containing high-glucose DMEM, 100 nM dexamethasone, 0.5 mM IBMX, and 50 µM IND (all Sigma-Aldrich). Cells were cultured in adipogenic media at 37 °C for 21 days and media changed every 3 days. To detect positive differentiation, cells were fixed with cold 4% paraformaldehyde for 12 minutes and stained with Oil Red O (Sigma-Aldrich) using standard protocols.

### hUC-MSC surface phenotyping assay

hUC-MSCs were cultured in xeno-free cell culture media on TCP. Cells were then detached with TrypLE and washed once with PBS. To minimize non-specific binding of antibodies, cells were incubated with 2% w/v bovine serum albumin (BSA) in PBS for 30 minutes. Cells were then aliquoted at concentrations of 3–5 × 10^5^/100 µL. One aliquot was reserved as an unstained control and those remaining were stained with the following antibodies: CD73, CD105, CD90, CD45, CD31, and HLA-DR, -DP, -DQ (Biolegend, San Diego, USA). Primary antibody was added to each aliquot to achieve a ratio of about 20:1 of cells in buffer to antibody. About 3–5 × 10^5^ cells were stained with saturating concentrations of (fluorophore)-conjugated antibodies. Cells were incubated in the dark on ice for 30 minutes. After incubation, cells were washed three times and then resuspended in 1X PBS and immediately analyzed by flow cytometry (Becton  Dickinson FACS Canto, BD Biosciences, Sparks, USA). Flow cytometer instruments were set using unstained cells. Cells were gated by forward versus side scatter to eliminate doublets. A minimum of 10,000 events was counted for each analysis.

### MHC II gene expression tests of cell sheets

Human bone marrow stem cells and adipose-derived stem cells were purchased from Lonza (USA). hUC-MSC, hBMSC and hADSC in passage 6 were seeded at cell numbers of 2 × 10^5^ cells/35-mm dish and cultured for 4–7 days to prepare cell sheets. hUC-MSC cell sheets were collected after detachment from TRCD at RT. Total RNA from cell sheets was extracted using trizol and PureLink RNA Mini Kt (Life Technologies, Carlsbad, USA) according to manufacturer’s protocols. cDNA was prepared from 1 μg of total RNA using high capacity cDNA reverse transcription kits (Life Technologies). RT-PCR analysis was performed with TapMan Universal PCR Master Mix using an Applied Biosystems Step One instrument (Applied Biosystems^TM^, Foster City, USA). Gene expression levels were assessed for the following genes: (1) glyceraldehyde 3-phosphate dehydrogenase (*GAPDH*, Hs99999905_m1) as a housekeeping gene, (2) human leukocyte antigen (HLA)-DRB (*HLA-DR*, Hs04192464_m1), (3) HLA-DPB (*HLA-DP*, Hs03045105_m1), and (4) HLA-DQB (*HLA-DQ*, Hs03054971_m1). All primers were manufactured by Applied Biosystems (sequences for each shown in Fig. 4). Relative gene expression levels were quantified by the comparative CT method^77^. Gene expression levels were normalized to GAPDH expression levels. Gene expression levels are relative to levels of the hADSC sheet group.

### hUC-MSC sheet preparation using different initial cell seeding and passage numbers

hUC-MSC sheets were prepared on TRCDs in various conditions including different initial cell seeding and passage numbers (Fig. 2). Passage 6 cells were seeded on 35-mm TRCDs (Cell Seed Ltd., Tokyo, Japan) at cell numbers of 5 × 10^4^ cells/dish, 1 × 10^5^ cells/35 mm dish and 2 × 10^5^ cells/dish. Passage 4–12 cells were seeded at a cell number of 2 × 10^5^ cells/dish. Fresh xeno-free cell culture media including 16.4 μg/mL of ascorbic acid (Sigma-Aldrich, St. Louis, USA) to make cell sheets was added at 1 day after seeding. Confluent cell sheets formed 4–6 days after seeding and were detached from TRCD at RT (Fig. 1). Cell morphologies were monitored using an AX10 microscope (Carl Zeiss Microimaging, Göttingen, Germany) with AxioVision software (Carl Zeiss Microimaging) before cell sheet detachment.

### Histological analysis

Cultured passage 6 cell sheets were removed from TRCD at RT and fixed with 4% PFA for 30 min and then embedded in paraffin. Embedded specimens were sectioned into 4 μm slices and stained with H&E, isotype immunoglobulin G (IgG) antibody (negative control), stem cell surface markers (CD44), ECM proteins (fibronectin; FN and laminin; LM) and cell-cell junctions (integrin-linked kinase; β-catenin). For fluorescence staining (IgG, CD44, FN, LM, and β-catenin), slides were immersed in antigen retriever solution (Sigma-Aldrich) for 20 min at 100 °C and washed with PBS 1X. Non-specific binding was blocked in PBS 1X containing 10% goat serum (Vector Laboratories, Burlingame, USA), for 1 h at room temperature. Primary antibody labeling (Abcam, Cambridge, USA) (1:100) at 4 °C proceeded overnight and then washed with PBS 1X. These specimens were treated with Alexa Fluor 594-conjugated secondary antibodies (Life Technologies) (1:200) for 1 h and mounted with ProLong Gold Antifade Reagent (Life Technologies). Immunofluorescence images were obtained using an AX 10 microscope (Carl Zeiss Microimaging) and analyzed with Axiovision software (Carl Zeiss Microimaging). For H&E staining, specimens were treated with hematoxylin solution (Sigma-Aldrich) for 3 min and subsequently with eosin solution (ThermoFisher Scientific, Kalamazoo, USA) for 5 min. The H&E stained specimens were dehydrated and mounted with Permount^TM^ (ThermoFisher Scientific, USA). H&E images were obtained using a BX 41 microscope (Olympus, Hamburg, Germany).

### Cell sheet microstructure observed using transmission electron microscopy

hUC-MSC sheets at passage 6 were fixed with a mixture of 2% paraformaldehyde, 2% glutaraldehyde, 0.1 M sodium phosphate buffer, and 2% osmium tetroxide (OsO_4_) in sodium phosphate buffer and dehydrated in a grade series of ethanol. Samples were then embedded in epoxy resin. Ultrathin sections (70 nm thickness) were observed with a transmission electron microscope (JEOL JEM1200EX, JEOL USA, Peabody, USA).

### Determination of hepatocyte growth factor (HGF) and tumor necrosis factor alpha (TNF-α) secretion from hUC-MSC sheets

hUC-MSC cell sheets at passage 6 were fabricated on TRCDs. Supernatant media over adherent cultured cells for 24 hours was collected just prior to cell sheet detachment from TRCD at RT. HGF and TNF-α amounts secreted from hUC-MSCs were measured by human HGF Quantikine ELISA and human TNF-α Quantikine ELISA kits, respectively (R&D Systems, Minneapolis, USA).

### Cell sheet placement into immune-deficient mice subcutaneous tissue

Six-week old immune-deficient mice (NOD.CB17-Prkdc^scid^/NCrCrl) (Charles River, San Diego, USA) were anesthetized by inhalation of 5% isoflurane. The percent isoflurane was decreased to 1–3% once the rodent was fully sedated. One small incision (approx. 3 cm) was made approximately near the center of the back and a standard subcutaneous dorsal pocket was generated using blunt dissection^78^. hUC-MSC (passage 6) cell sheets (1 cm diameter circles) were detached from TRCD at RT after 4 days of culture and transplanted into subcutaneous dorsal tissue pockets^78^. Sterilized non-cytotoxic silicone membranes (a rectangle with length 1.2 cm, width 1.2 cm, and 0.3 mm thickness) (Invitrogen) were placed between each implanted cell sheet and subcutaneous dorsal tissues to block tissue adhesion. The incision was closed with a simple interrupted pattern using absorbable thread. Implanted mice (n = 6) were sacrificed at 10 days after cell sheet transplantation. The cell sheet-transplanted subcutaneous tissue was harvested and fixed with 10% formaldehyde (Sigma-Aldrich) for 1 day for histological analysis (see Materials and Methods; 2.7. Histological analysis). All procedures were approved by the Institutional Animal Care and Use Committee (IACUC) (protocol #16–12017) at The University of Utah and conducted in accordance with accepted international guidelines.

### Statistical analyses

All quantitative values are expressed as mean and standard error (SE, mean ± SE). Significant differences between groups were tested by one-way Analysis of Variance using Origin 2017 software (OriginLab, Northampton, USA). A probability value of less than 0.05 (*p* < 0.05) was considered statistically significant.

## Supplementary information

Supplementary Information
