# Supplementary material for: Human mesenchymal stem cell sheets in xeno-free media for possible allogenic applications
Source: Sci Rep. 2019 Oct 8;9:14415. doi: 10.1038/s41598-019-50430-7 (PMC6783458; doi:10.1038/s41598-019-50430-7)

Human mesenchymal stem cell sheets in xeno-free media for possible allogenic applications

Kyungsook Kim[1,*], Sophia Bou-Ghannam[1], Hallie Thorp[1],

David W. Grainger[1], Teruo Okano[1,2,*]

[1]*Cell Sheet Tissue Engineering Center (CSTEC), Department of Pharmaceutics and Pharmaceutical Chemistry, Health Sciences, University of Utah, 30 South 2000 East, Salt Lake City, Utah 84112 USA*

[2]*Institute of Advanced Biomedical Engineering and Science, Tokyo Women's Medical University, 8-1 Kawada-cho, Shinjuku-ku, Tokyo 162-8666, Japan*

*Corresponding authors: Kyungsook Kim, Ph.D. and Teruo Okano, Ph.D.

Cell Sheet Tissue Engineering Center (CSTEC), Department of Pharmaceutics and Pharmaceutical Chemistry, Health Sciences, University of Utah, 30 South 2000 East, Salt Lake City, Utah 84112 USA

Tel: +1-801-585-0070

Fax: +1-801-581-3674

E-mail: kyungsook.kim@utah.edu

Supplemental results

Cell junction proteins of hUC-MSC sheet

To verify cell junction protein gene expression levels, quantitative RT-PCR analysis was performed to measure integrin-linked kinase (ILK) and N-cadherin (N-cad) gene expression levels of hUC-MSCs in different passage numbers. Passage 12 cell showed lower ILK and N-cad gene expression, compared to passage 6, 8, and 10, indicating that hUC-MSCs in passage 12 have insufficient cell junction proteins compared to these cells from smaller passage numbers in Figure S1.

hUC-MSC multi-potency to chondrocytes

hUC-MSC differentiation potential into chondrocytes were characterized for passage 6, 8, 10, and 12, compared with control group in Figure S2. hUC-MSC showed low positive expression in Alcian blue staining. There was no differences between differentiated and control groups.

Secretion of vascular endothelial growth factor (VEGF)

To verify vascularization ability of cell sheets, quantitative RT-PCR analysis was performed to measure human VEGF gene expression, which is a key contributor in the formation of new blood vessels. hVEGF gene expression in hUC-MSC, hBMSC, and hADSC sheets were compared. hUC-MSC, hADSC, and hBMSC showed hVEGF gene expression. There was no significant differences of hVEGF gene expression levels in all groups (Figure S3).

Supplemental materials and methods

Quantitative reverse transcription polymerase chain reaction (RT-PCR) analysis

hUC-MSC cell sheets were collected after detachment from TRCD at RT. Total RNA from cell sheets was extracted using Trizol and PureLink RNA Mini Kt (Life Technologies) according

to manufacturer's protocols. cDNA was prepared from 1 μg of total RNA using high capacity cDNA reverse transcription kits (Life Technologies). RT-PCR analysis was performed with TaqMan Universal PCR Master Mix using an Applied Biosystems Step One instrument (Applied Biosystems, Foster City, USA). Gene expression levels were assessed for the following genes: 1) glyceraldehyde 3-phosphate dehydrogenase (*GAPDH*, Hs02786624_g1) as a housekeeping gene, 2) integrin-linked kinase (*ILK*, Hs00177914_m1), 3) N-cadherin (*N-cad*, Hs00983056_m1), 4) vascular endothelial growth factor (*VEGF*, Hs99999070_m1) All primers were manufactured by Applied Biosystems (sequences for each shown in Table S1, Supplementary data). Relative gene expression levels were quantified by the comparative C_T method [77]. Gene expression levels were normalized to GAPDH expression levels. Gene expression levels are relative to levels for the passage 6 cell group.

hUC-MSC differentiation potential to chondrocytes

For chondrogenic differentiation, cells were aliquoted at 2.5 x 10^5 cells in 15 mL tubes and centrifuged at 12000 rpm for 10 min. Tubes were then incubated at 37°C for 3 days with loosened caps to allow pellet formation. After 3 days, media was changed to StemPro chondrogenic induction media (Life Technologies) and pellets were incubated in chondrogenic induction media at 37°C for 3 weeks, with media changed twice per week. At 3 weeks, pellets were fixed with cold 4% Paraformaldehyde for 12 mins and paraffin embedded. To detect positive differentiation, 4μm paraffin sections were then stained with Alcian blue (Sigma-aldrich) for 10 min according to standard protocols.

Supplemental figure captions

Supplemental figure 1. Cell-cell junction related gene expression levels from hUC-MSC sheets. Gene expression levels of (a) integrin-linked protein kinase (ILK) and (b) N-cadherin (Ncad) associated with cell junctions in passage 12 were lower than that in passage 6.

Supplemental figure 2. hUC-MSC differentiation potential. hUC-MSCs (passages 6-12) were differentiated into (b) chondrocytes. (a) Control pellets were cultured in normal cell culture media for 3 weeks in 15 ml tubes. Scale bars are 20 μm.

Supplemental figure 3. Human vascular endothelial growth factor (VEGF) gene expression levels of hUC-MSC, hBMSC, and hADSC sheets in passage 6. All groups are not significantly different.

Supplemental figure 1

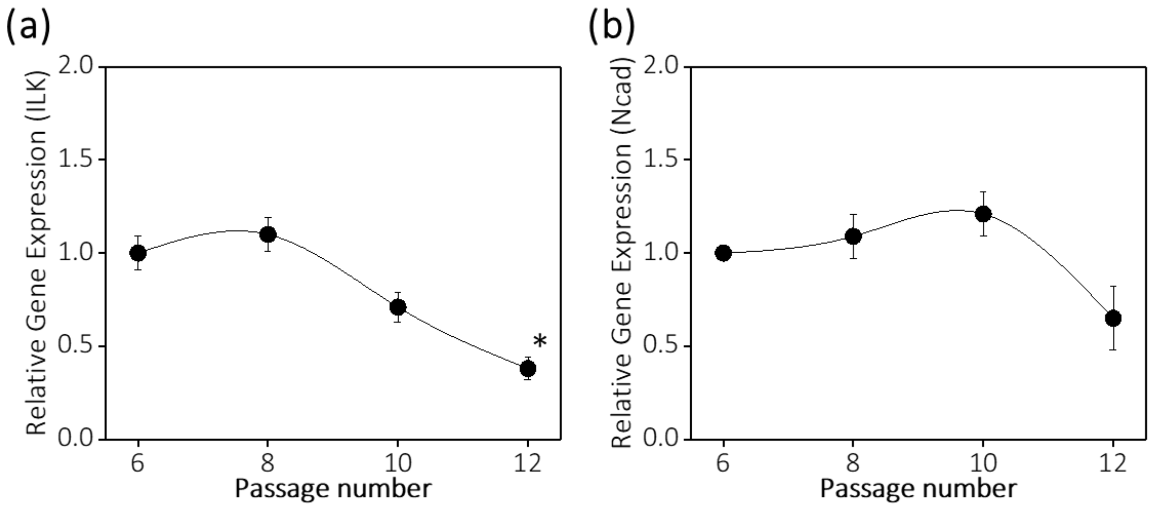

Supplemental figure 2

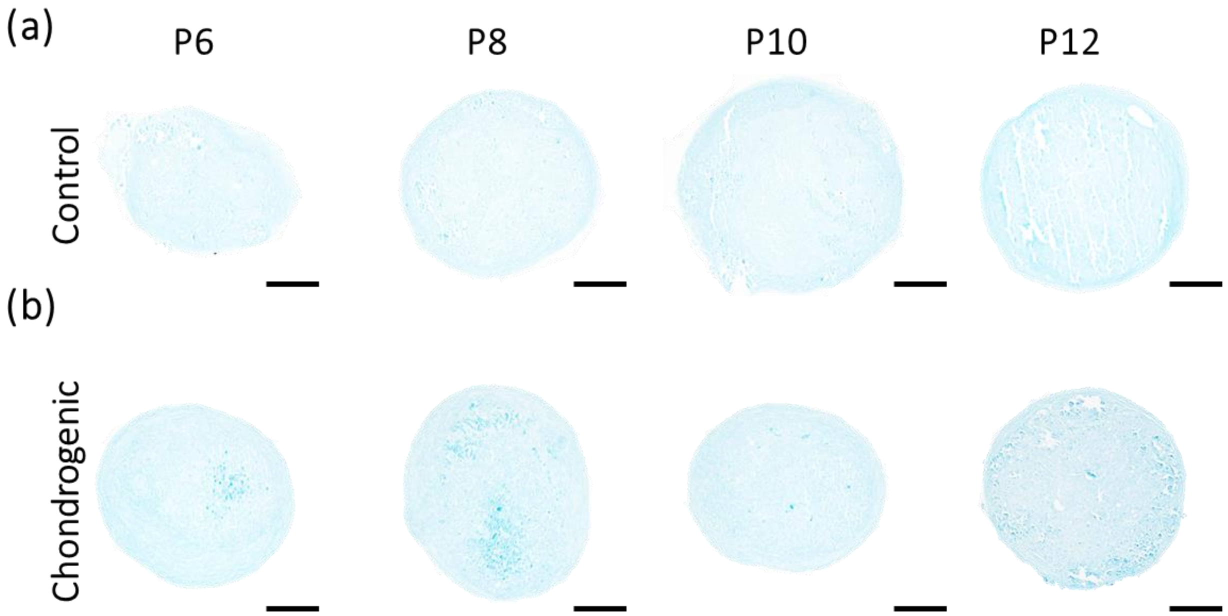

Supplemental figure 3

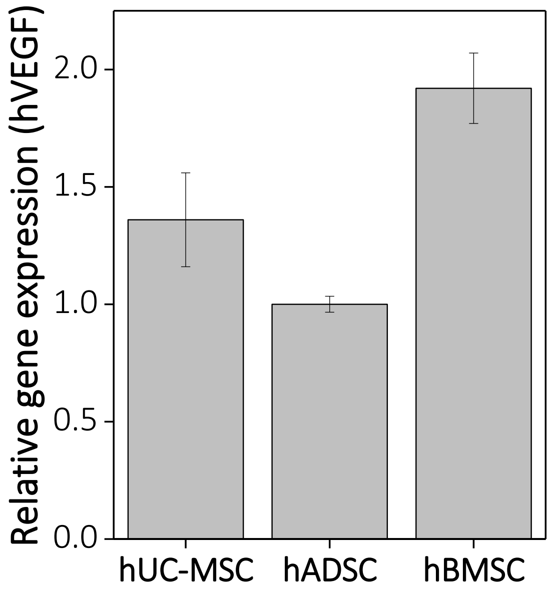

Supplement: Supplementary file 1 — Supplementary Information [file 41598_2019_50430_MOESM1_ESM.pdf]
